# Telomerase Does Not Improve DNA Repair in Mitochondria upon Stress but Increases MnSOD Protein under Serum-Free Conditions

**DOI:** 10.3390/ijms21010027

**Published:** 2019-12-19

**Authors:** Alexander Martens, Bianca Schmid, Olasubomi Akintola, Gabriele Saretzki

**Affiliations:** The Ageing Biology Centre, Biosciences Institute, Campus for Ageing and Vitality, Newcastle University, Newcastle upon Tyne NE4 5PL, UK

**Keywords:** telomerase, mitochondria, DNA damage, DNA repair MnSOD, antioxidants

## Abstract

Telomerase is best known for its function in maintaining telomeres but has also multiple additional, non-canonical functions. One of these functions is the decrease of oxidative stress and DNA damage due to localisation of the telomerase protein TERT into mitochondria under oxidative stress. However, the exact molecular mechanisms behind these protective effects are still not well understood. We had shown previously that overexpression of human telomerase reverse transcriptase (hTERT) in human fibroblasts results in a decrease of mitochondrial DNA (mtDNA) damage after oxidative stress. MtDNA damage caused by oxidative stress is removed via the base excision repair (BER) pathway. Therefore we aimed to analyse whether telomerase is able to improve this pathway. We applied different types of DNA damaging agents such as irradiation, arsenite treatment (NaAsO_2_) and treatment with hydrogen peroxide (H_2_O_2_). Using a PCR-based assay to evaluate mtDNA damage, we demonstrate that overexpression of hTERT in MRC-5 fibroblasts protects mtDNA from H_2_O_2_ and NaAsO_2_ induced damage, compared with their isogenic telomerase-negative counterparts. However, overexpression of *hTERT* did not seem to increase repair of mtDNA after oxidative stress, but promoted increased levels of manganese superoxide dismutase (MnSOD) and forkhead-box-protein O3 (FoxO3a) proteins during incubation in serum free medium as well as under oxidative stress, while no differences were found in protein levels of catalase. Together, our results suggest that rather than interfering with mitochondrial DNA repair mechanisms, such as BER, telomerase seems to increase antioxidant defence mechanisms to prevent mtDNA damage and to increase cellular resistance to oxidative stress. However, the result has to be reproduced in additional cellular systems in order to generalise our findings.

## 1. Introduction

Telomerase is best known for its role in telomere maintenance. However, various non-telomeric functions have been described for the catalytic subunit TERT (Telomerase Reverse Transcriptase) (for review see [1]). One of these non-canonical functions of TERT is the shuttling of the TERT protein from the nucleus to mitochondria under oxidative stress [2,3,4,5]. Several studies demonstrated a protective function of telomerase/TERT on mitochondrial DNA after various stress types [3,4], as well as after transfection of a TERT mutant with a defective nuclear exclusion site (NES) resulting in an inability to leave the nucleus [6,7]. However, the exact molecular mechanisms behind the observed protective effects still remain largely elusive. While some authors reported an improved respiration, complex I and cytochrome c oxidase activity [3,8], others found an increased antioxidant defence in *hTERT* overexpressing cells [8,9].

Thus, we aimed to address whether mitochondrial TERT which is localised to mitochondria quickly after an acute treatment with hydrogen peroxide (H_2_O_2_) or irradiation, rather prevents the initial mitochondrial (mt) DNA damage or rather promotes a better DNA repair as had been hypothesised by previous studies for nuclear DNA [9]. We had found previously that mitochondrial localisation of TERT protein correlates with lower amounts of nuclear DNA damage after H_2_O_2_ treatment and γ-irradiation, resulting in less apoptosis [5]. Our lab and others had also demonstrated that mitochondrial TERT localisation is associated with lower reactive oxygen species (ROS) levels in different cellular models [2,3,5,8]. We use here an hTERT-overexpressing cell model on the basis of human fibroblasts MRC-5 which we had characterised in detail previously [3,5,10]. Although *hTERT* overexpression in normal fibroblasts devoid of the gene expression and telomerase activity might not be physiologically relevant, it can reflect events that occur during tumourigenesis when telomerase activity is acquired and strongly upregulated.

There are multiple mtDNA copies per mitochondrium with a variable amount of mitochondria per cell, depending on cell type. Human mitochondrial DNA is a double stranded circular molecule of 16.569 base pairs encoding various transport and ribosomal RNAs as well as 13 proteins involved in oxidative phosphorylation and cellular energy production. Since mtDNA lacks histones and is generally less well repaired than genomic nuclear DNA, mutation frequency of mitochondrial DNA is believed to be substantially higher [11].

Consequently, maintenance of mitochondrial DNA integrity is essential for cellular function. Mitochondrial DNA damage by oxidative stress is known to be removed by the base excision repair (BER) pathway. Although various DNA repair mechanisms have been found and described within mitochondria, BER is the best studied one [12]. However, there are different mechanisms to protect mitochondrial DNA including various antioxidant enzymes such as manganese superoxide dismutase (MnSOD), glutathione, thioredoxins, peroxiredoxins and many more [13].

Our study used a PCR-based technique to specifically measure mitochondrial DNA damage after various types of stress (irradiation, arsenite and hydrogen peroxide). While irradiation did not generate any mtDNA damage, arsenite damaged mtDNA in parental fibroblasts predominantly without any measurable repair. Treatment with 200 µM hydrogen peroxide induced higher DNA damage immediately after 1 h of treatment in parental MRC-5 cells compared to *hTERT* overexpressing cells, but no difference in DNA repair was found over the course of 48 h post treatment between the two cell types. Interestingly, we found a significant increase in MnSOD protein level in *hTERT* overexpressing fibroblasts during H_2_O_2_ treatment and serum-withdrawal which correlated to higher protein levels of forkhead-box-protein O3 (FoxO3a), a known upstream regulator of MnSOD although no transcriptional upregulation of MnSOD was found.

## 2. Results

**Figure 1:**
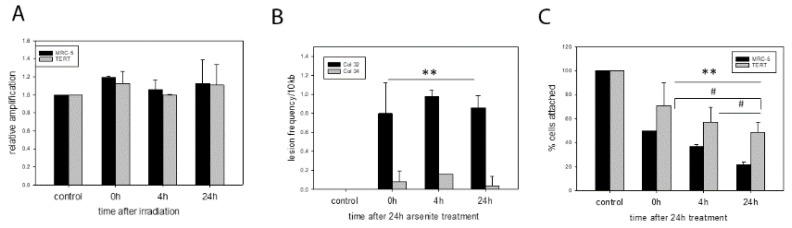
mtDNA damage in MRC-5 and *hTERT* overexpressing cells after x-irradiation and arsenite treatment. (**A**) relative amplification of parental and *hTERT* overexpressing MRC-5 fibroblasts for untreated cells (control) as well as immediately (0 h), 4 h and 24 h after irradiation. (**B**) Mitochondrial DNA damage measured as lesion frequency per 10 kb in parental and *hTERT* overexpressing MRC-5 cells at different time points (0 h, 4 h, 24 h) after 24 h treatment with 50 μM sodium arsenite. Control = untreated. A general linear model statistics found that lesion frequency in MRC-5 cells is significantly different from that in *hTERT* overexpressing cells (** *p* = 0.002) while there are no significant differences between the different time points. (**C**) Percentage of cells attached to the culture flask for untreated cells (control) and the indicated time points after 24 h treatment with 50 μM Sodium arsenite. A general linear model statistics found that the number of attached cells in MRC-5 cells is significantly different from *hTERT* overexpressing cells (** *p* = 0.008). For the different time points there were significant differences comparing 0 h and 24 h (^#^ *p* = 0.029), 4 h and 24 h (^#^ *p* = 0.04) while 0 h versus 4 h was not significant (*p* = 0.068). Data represent mean values with error bars ± SD of at least two independent experiments (*n* = 2).

Surprisingly, we did not find any mtDNA damage or lesions after x-ray irradiation at relatively high doses such as 20 Gy. Due to the absence of any lesions we show the relative amplification efficiency which is 100% at all time points after the initial irradiation (Figure 1A). MtDNA damage levels were expressed as the relative amplification of the 11 kb long fragment, normalized against the 83 bp fragment (see methods). A 20 Gy dose is normally able to induce senescence in MRC-5 fibroblasts [14,15] and induces genomic damage immediately after irradiation while driving TERT protein into mitochondria [5]. This mitochondrial localisation of TERT is able to counteract or even prevent irradiation-induced genomic DNA damage and downstream apoptosis [5].

In contrast, we observed different amounts of mtDNA damage in the two cell types after exposure of cells to 50 μM sodium arsenite for 24 h. While parental MRC-5 fibroblasts had a lot of DNA damage present at all 3 time points after a 24 h treatment period, there was hardly any DNA damage (measured as lesion frequency per 10 kb) detectable in *hTERT* over-expressing fibroblasts (Figure 1B). Interestingly, no DNA repair was found even 24 after removal of the arsenite from the cell culture. The effect of arsenic also impacted the number of attached cells over the observed time period after sodium arsenite treatment. Cell numbers also decreased in *hTERT* over-expressing cells, most likely due to additional effects on nuclear DNA, but there was a much steeper decline in cell numbers for the MRC-5 cells (Figure 1C) correlating to the high amount of mitochondrial DNA damage.

**Figure 2:**
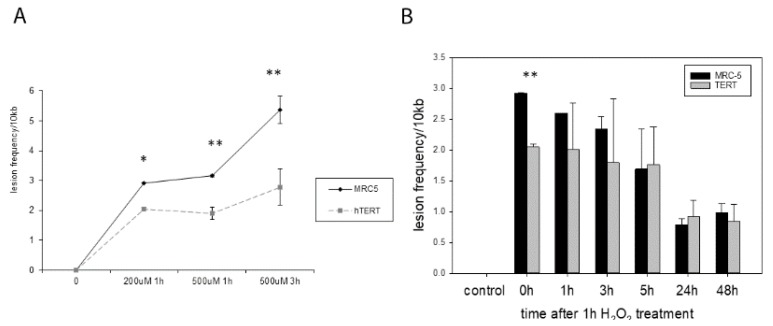
mtDNA damage in MRC-5 and *hTERT* overexpressing cells with different concentrations of hydrogen peroxide. (**A**) Mitochondrial DNA damage measured as lesion frequency per 10 kb in parental and *hTERT* overexpressing MRC-5 cells at different time points (0 h, 1 h, 3 h) after treatment with either 0 (control), 200 µM or 500 µM of H_2_O_2_ as indicated. An unpaired *t*-test was performed for each of the treatments and significance determined: 1 h 200 µM H_2_O_2_ (* *p* = 0.02), 1 h 500 µM of H_2_O_2_ (^**^ *p* = 0.0012) and 3 h 500 µM H_2_O_2_ (** *p* = 0042). (**B**) Mitochondrial DNA damage measured as lesion frequency per 10 kb in parental and *hTERT* overexpressing MRC-5 cells at different time points (0 h, 1 h, 3 h, 5 h, 24 h and 48 h) after 1 h treatment with 200 µM H_2_O_2_ in order to study DNA repair. Only the time point immediately after the treatment was significant in an unpaired t-test (** *p* = 0.0019). Data represent mean values with error bars ± SD of at least two independent experiments (*n* = 2).

We know from previous studies that *hTERT* overexpressing cells show less mtDNA damage immediately after hydrogen peroxide treatment [2]. We initially reproduced these results here for treatment with 200 µM H_2_O_2_ measured immediately after 1 h treatment as well as with 500 µM H_2_O_2_ after 1 h and 3 h of treatment (Figure 2A). While there were no significant differences found using a multiple comparison test, analysing the differences in lesion frequency between MRC-5 and *hTERT* overexpressing cells separately for each condition, a significant difference was found in an unpaired t-test for all different treatment conditions.

We were then interested to analyse whether *hTERT* overexpression also improves DNA repair as it had been suggested for genomic DNA [9]. Treatment with 200 µM H_2_O_2_ for 1 h generated a significantly different amount of mtDNA damage in the two cell types when analysed immediately after the treatment (0 h, *p* = 0.0019 Figure 2B). We found that the majority of mtDNA damage was repaired after 24 and 48 h, however, there was no difference in repair capacities between the two cell types.

**Figure 3:**
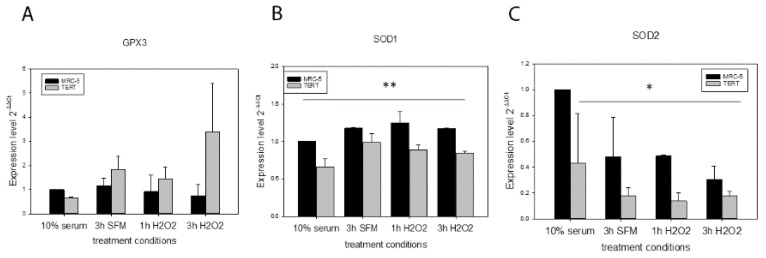
Expression analysis of antioxidant genes in MRC-5 and *hTERT* overexpressing cells after treatment with 200 µM H_2_O_2_. (**A**) *GPX3* expression in parental and *hTERT* overexpressing MRC-5 cells for the indicated treatment conditions. (**B**) *SOD1* expression in parental and *hTERT* overexpressing MRC-5 cells for the indicated treatment conditions. A general linear model statistics found that *SOD1* expression in MRC-5 cells is significantly higher than that in *hTERT* overexpressing cells (** *p* = 0.004). (**C**) *SOD2* expression in parental and *hTERT* overexpressing MRC-5 cells for the indicated treatment conditions. A general linear model statistics found that *SOD2* expression in MRC-5 cells is significantly higher than that in *hTERT* overexpressing cells (* *p* = 0.035). Data represent mean values with error bars ± SD of at least two independent experiments (*n* = 2).

In order to explore alternative mechanisms of how *hTERT* overexpression could improve resistance against initial mtDNA damage we analysed the gene expression of different antioxidant enzymes such as GPX3, SOD1 and SOD2 under control conditions (medium containing 10% FCS), 3 h serum free medium (SFM) used for the H_2_O_2_ treatment as well as 1 h and 3 h of treatment with 200 µM H_2_O_2_. While we did not find any differences for *GPX3* expression (Figure 3A), there was significantly less *SOD1* and *SOD2* expression in *hTERT* overexpressing cells compared to MRC-5 parental fibroblasts in most conditions analysed (Factor “cell type” in a general linear model statistics, Figure 3B,C).

**Figure 4:**
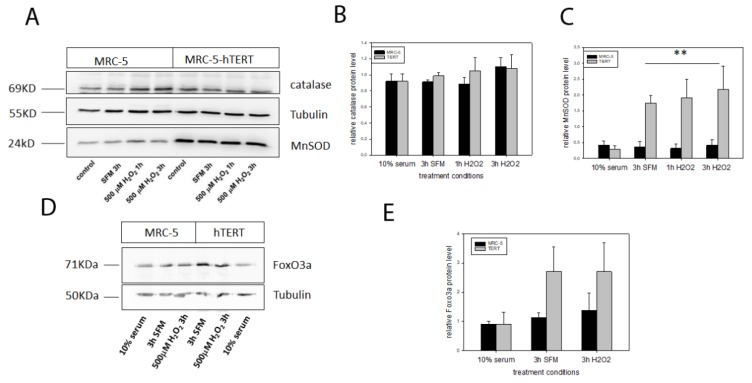
Western Blot (WB) analysis of protein levels of catalase, MnSOD and Foxo3a in MRC-5 and *hTERT* overexpressing cells. (**A**) Representative WB for catalase, MnSOD and tubulin in control cells in 10% serum containing medium, 3 h serum free medium (SFM) conditions and 1 h and 3 h treatment with 500 µM H_2_O_2_. (**B**) Quantification of catalase protein level relative to tubulin protein. (**C**) Quantification of MnSOD protein level relative to tubulin protein. A general linear model statistics for the conditions excluding the full serum medium found that MnSOD protein level in *hTERT* overexpressing cells is significantly higher than that in MRC-5 cells (** *p* = 0.005). (**D**) Representative WB for Foxo3a under the indicated conditions (**E**) FoxO3a protein level relative to tubulin for full serum medium, SFM and 3 h treatment with 500 µM H_2_O_2_. The data is not statistically significant between cell types. Data represent mean values with error bars ± SD of at least two independent experiments (*n* = 2).

Since gene expression is not always representative for antioxidant enzymes and their activity we also analysed the mitochondrial MnSOD as well as catalase at the protein level using Western Blot analysis for the same conditions as for the expression analysis (Figure 4A). While there were no differences for levels of catalase protein for any of the used conditions (Figure 4B), there was a significant increase in MnSOD protein level in *hTERT* overexpressing cells under serum-free conditions with no significant further increase under H_2_O_2_ treatment (Figure 4C). Since MnSOD is a downstream target of Foxo3a, an important transcription factor involved in stress response and longevity (reviewed in [16]) we also analysed protein levels of Foxo3a in MRC-5 cells and their *hTERT* overexpressing counterparts. Foxo3a protein levels were similar for both cell types under 10% serum in the medium. In contrast, although not statistically significant (neither using a two-way ANOVA nor a *t*-test for each condition separately), there was a tendency towards increased Foxo3a levels for *hTERT* overexpressing cells under serum-free conditions that did not increase further after 3h treatment with 500 µM H_2_O_2_ (Figure 4D,E) corresponding to increases in MnSOD under the same conditions.

**Figure 5:**
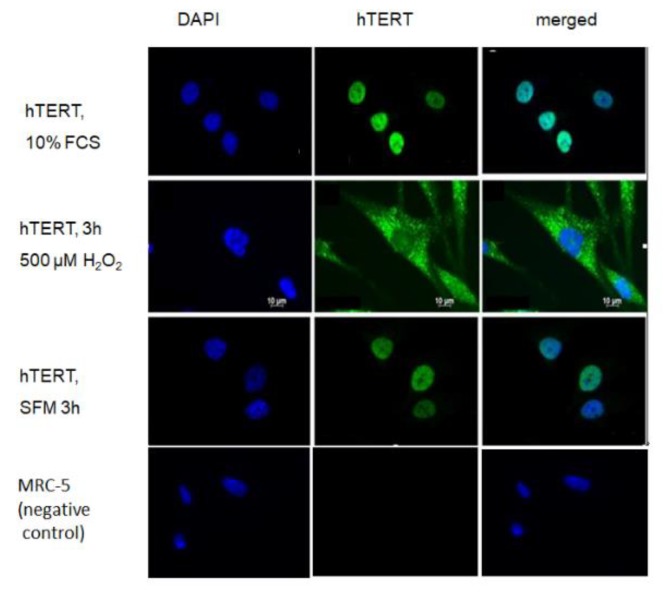
Subcellular localisation of hTERT protein in *hTERT* overexpressing cells under basal condition (full serum medium), 3 h treatment with 500 µM H_2_O_2_ and 3 h serum free medium (SFM). Representative images. Left column: DAPI staining, middle column: hTERT staining, right column: merged staining. While the upper and third rows show exclusively nuclear hTERT staining the second row where H_2_O_2_ had been applied shows a predominant cytoplasmic staining of hTERT which to a certain degree is presumed to localise within mitochondria as demonstrated previously [2,5]. The specificity of the used anti-hTERT antibody is shown using MRC-5 cells that are negative for hTERT staining as already demonstrated previously for this antibody [18].

Since it is well known that mitochondrial localisation of telomerase is triggered by oxidative stress we examined whether treatment of hTERT-overexpressing cells with serum free medium alone poses a stress that is able to drive hTERT protein from the nucleus into mitochondria where it could possibly directly interact with the MnSOD protein. While H_2_O_2_ treatment indeed excluded hTERT protein from the nucleus as demonstrated previously [2,5,17], the serum-free condition was not able to exclude hTERT from the nucleus and was indistinguishable from serum-containing media condition (Figure 5).

## 3. Discussion

The aim of our study was to examine whether the protective effect of hTERT also includes an improved mitochondrial DNA repair as it was shown for nuclear DNA [9]. In addition, we had demonstrated previously that mitochondrial hTERT localisation correlates to less nuclear DNA damage [5]. To achieve our aim we used different types of treatment. Ionizing irradiation induces various cellular effects by either directly interacting with DNA or by forming hydroxyl radicals, which can further damage DNA [19]. Surprisingly, 20 Gy of x-irradiation did not generate any mtDNA breaks in our hands. The absence of DNA damage could be due to the small size of the mitochondrial genome. X-ray irradiation is known to generate mainly strand breaks (single and double stranded), but that could be a rather infrequent event which only matters in a large genome such as the nuclear [20]. This suggestion is in agreement with experiments from Spangler and co-authors [21] who found that DNA damage from γ-irradiation was most consistent with a single-hit mechanism and did not differ in DNA fragments of different size; again suggesting it to be a rather rare event. DNA repair in mtDNA after high doses of irradiation has not been investigated very intensely, most studies instead focused on mutation rates, changes in mtDNA copy numbers [22,23] or found a relaxation in supercoiling of the double stranded mitochondrial DNA [24]. Others pointed out that the formation of mitochondrial deletions took time after the initial irradiation event since it required mtDNA replication to occur [25]. May and Bohr [26] have even used 560 Gy in order to detect DNA damage in mitochondrial DNA. Although irradiation also induces ROS, the high amount of irradiation might preferentially induce double strand breaks (DSB) which are predominantly repaired by non-homologous end joining (NHEJ), a repair mechanism not found in mammalian mitochondria [27].

In contrast, we found substantial differences between the two cell types after 24 h of 50 µM arsenite treatment: while there was not much mtDNA damage in *hTERT*-overexpressing cells, MRC-5 fibroblasts were rather sensitive against arsenite treatment. Arsenite is an important environmental pollutant of drinking water and known to generate mitochondrial ROS and to induce oxidative damage in mtDNA [28,29]. It is a potent mutagen and its effects are thought to be mediated predominantly through reactive oxygen species. Our findings are consistent with those of Kessel and colleagues [28] and Liu and co-authors [29] who demonstrated that mitochondria are a primary target in arsenic-induced mutagenicity and genotoxicity. However, there was no meaningful repair observed until 24 h after the treatment stopped. This could be due to the fact that arsenite is also known to inhibit DNA repair, for example via indirectly inhibiting DNA ligase [30] which is an important enzyme during BER. Ebert and colleagues [31] showed that arsenic from 5 µM onwards can also inhibit human 8-oxoguanine DNA glycosylase-1 (OGG1) activity. In our experiments arsenite treatment also impacted on the number of cells attached onto the culture plates. In both cell types cell numbers decreased over the observation time which could be due to ongoing apoptosis as well as effects to other cellular compounds including nuclear DNA. However, corresponding to less mtDNA damage cell numbers were higher in *hTERT*-expressing cells than parental fibroblasts. Thus, it seems that the presence of hTERT reduced oxidative stress as shown previously for acute and chronic stress treatments [3].

As expected from our previous study [3] we confirmed differences in initial mtDNA damage levels between parental and *hTERT* overexpressing fibroblasts after treatment with 200 µM H_2_O_2_ for 1 h. However, following mtDNA damage levels until 48 h after initial damage, we could not identify any superior DNA repair due to the presence of telomerase. MtDNA damage from oxidative stress is usually repaired by base excision repair (BER) and it seems that the presence of hTERT does not interfere with this repair mechanism within mitochondria.

Instead, we found an increased amount of mitochondrial MnSOD protein in *hTERT* overexpressing cells. However, in contrast to Sharma and co-authors [9] we did not detect an upregulation of *SOD2/MnSOD* on the gene expression level in *hTERT*-overexpressing cells and also not for *SOD1* or *GPX3*. Due to the fact that *SOD1* and *SOD2* expression levels are even lower in *hTERT*-expressing cells we speculate that possibly either the mRNA or the MnSOD protein stability might be increased in those cells.

Surprisingly, MnSOD was not only upregulated by increased stress, but already by serum withdrawal. In order to examine whether serum-free medium could exclude hTERT protein from the nucleus, we analysed hTERT protein localisation under different treatment conditions and, as demonstrated previously [2,5], we found nuclear hTERT exclusion after H_2_O_2_ treatment while serum free medium did not result in any changes in hTERT localisation compared to medium containing 10% serum. Consequently, the increase in MnSOD protein was unlikely related to hTERT localisation within mitochondria, for example by directly stabilising the protein, but most likely due to an interaction of hTERT with signalling pathways induced under serum conditions, such as increased Foxo3a signalling. Indeed, we identified higher amounts of the stress-related transcription factor Foxo3a protein which is an upstream regulator of MnSOD. This result suggests that hTERT protein might induce (directly or indirectly via upstream components in the signalling cascade) Foxo3a protein and its downstream target MnSOD under serum-free conditions.

Others have shown previously that FoxO3a is increased by serum deprivation [32] and that MnSOD protects quiescent cells via FoxO3a [33]. Kops and colleagues [32] demonstrated induction of MnSOD in 3T3 cells via transcriptional regulation of gene expression after several hours. In contrast, our data did not confirm an increased *MnSOD* gene expression while protein levels were increased in serum free medium around five-fold but did not increase any further by increased oxidative stress. This result suggests that the changes might have occurred rather on a post-transcriptional level. Although we have used only one isogenic cell pair here our results of an increased level of antioxidants after *hTERT* overexpression are in accordance with findings from others who have demonstrated that the antioxidant effect of hTERT correlated with an increase in the ratio of reduced to oxidized glutathione (GSH:GSSG) together with higher glutathione peroxide (GPx) and glutathione reductase (GR) activities as well as improved recovery of oxidised peroxiredoxin to its non-oxidised form in HeLa cells overexpressing hTERT [8]. Although it is desirable to validate our finding in other cell models, it is quite possible that different genetic make-ups of cell lines might generate variable results due to the plethora of different functions and interactions of hTERT and telomerase with other cellular components and pathways (for review, see [1]). Together, our finding of a higher MnSOD protein level adds to previous descriptions of a potential improvement of different enzymes and factors of the antioxidant response by hTERT in different cell models.

## 4. Material and Methods

### 4.1. Buffers and Solutions

All chemicals and solutions were obtained from Sigma-Aldrich UK, if not described otherwise.

### 4.2. General Tissue Culture Conditions

Human embryonic lung MRC-5 fibroblasts were obtained from ECACC (Salisbury, UK) and used at population doubling levels (PDL) of 25–35. These cells were retrovirally transfected with the human catalytic subunit of the enzyme telomerase (hTERT) previously [3] and used as isogenic controls that are telomerase positive. These cells were used at PDLs of 125–165. All cells were cultured in DMEM with high glucose and L-glutamine (PAA Laboratories, Austria), plus 10% FCS, 1% penicillin–streptomycin (PAA Laboratories, Pasching, Austria) and 2 mM L-glutamine (PAA Laboratories, Pasching, Austria) in a cell culture incubator (Thermo Forma, Marietta, OH, USA) at 37 °C, 62% relative humidity and 5% CO_2_. Cells were seeded and cultured for at least 16 h before any treatment.

### 4.3. Treatments of Cells

#### 4.3.1. X-Ray Irradiation of Cells

Irradiation experiments were performed using a CP-160 Cabinet X-Radiator™ System (Faxitron, Lincolnshire, IL, USA) by exposing the cells to 20 Gy. After the irradiation culture medium was renewed and cells were allowed to grow further for the indicated time points or trypsinised immediately. Pellets were stored at −80 °C until DNA was extracted.

#### 4.3.2. Sodium Arsenite Treatment

Cells were treated with 50 µM NaAsO_2_ for 24 h in full medium. After the treatment, the cells were washed with PBS and trypsinised immediately or fresh culture medium was added and cells were further grown until cell pellets were generated and stored at −80 °C.

#### 4.3.3. Treatment with H_2_O_2_

Wild-type MRC-5 and *hTERT* overexpressing fibroblasts were seeded in 25 cm^2^ tissue culture flasks and washed with PBS before treatment with serum free medium (SFM) containing 0, 200 or 500 μM H_2_O_2_ for 1 h or 3 h. SFM was used to avoid inactivation of H_2_O_2_ by serum. After incubation the H_2_O_2_ was removed and inactivated by adding serum containing DMEM. The cells were then washed with PBS and trypsinised immediately with 1xTrypsin-EDTA or fresh culture medium was added and cells were allowed to recover for different periods of time. Cell pellets were kept at −80 °C until they were used for DNA or protein extraction.

### 4.4. Mitochondrial DNA Damage Assay

Mitochondrial DNA damage assay was performed as described earlier [2,3] and is described here briefly:

#### 4.4.1. DNA Isolation

Whole genomic DNA from frozen pellets was isolated using the QIAamp DNA Mini Kit (QIAGEN, Hilden, Germany) according to the manufacturer’s instructions.

#### 4.4.2. Amplification of an 83 bp Amplicon

As the mitochondrial copy number can vary from cell to cell, the amount of mtDNA has to be normalised. MtDNA concentration was standardised by amplifying a short 83 bp fragment together with the created standard dilutions of the control template, which had a known relative copy number. The standardisation of the samples was performed by using the SYBR^®^ Green JumpStart™ Taq ReadyMix™ for Quantitative PCR (Sigma©-Aldrich, UK) in 25 μL reactions with 0.4 μM primers (5′-GAT TTG GGT ACC ACC CAA GTA TTG-3′ (IS1) and 5′-AAT ATT CAT GGT GGC TGG CAG TA-3′ (IS2), as previously described [3] and 60 ng template DNA. To normalise all samples and compensate for potential inequalities between samples, 1× ROX passive reference dye was added. Reactions were performed on a StepOnePlusTM Real Time PCR System (Applied Biosystems, Warrington, UK). PCR conditions were: 6 min at 94 °C, then 35 cycles with 94 °C for 15 s, 60 °C for 45 s and 72 °C for 45 s. Melting curve analysis was performed after each run between 60 and 95 °C in 1 °C intervals.

#### 4.4.3. Amplification of the 11,009 bp Amplicon

The PCR reactions for the 11,009 bp fragment were performed under the following conditions: an initial heat denaturation step for 6 min at 94 °C, followed by 35 cycles of 94 °C for 15 s, 8.5 min at 68 °C with additional 10 s after each cycle and 80 °C for 10 s. At the end of each run melting curve analysis was performed with measurements at 1 °C intervals between 60 °C and 95 °C. To detect the amplification in real time, 1 μL 5× SYBR^®^ Green I in DMSO (Rockland Immunochemicals, Philadelphia, PA, USA) was added to each reaction. Instead of using 1 μL (60 ng) template DNA, samples were standardised to 1 × 10^6^ copies/μL, after having assessed the relative copy-number in each sample via the amplification of the 83 bp fragment. 1× ROX Reference dye was added to each sample. Primer Sequences were OLA: 5′-GGG AGA AGC CCC GGC AGG TTT GAA GC-3′ and D1B: 5′-ATG ATG TCT GTG TGG AAA GTG GCT GTG C-3′. Reactions were performed on a StepOnePlusTM Real Time PCR System (Applied Biosystems, Warrington, UK). The entire PCR product was run a 0.8% TBE agarose gel containing EtBr, using a 6× Blue/Orange Loading Dye (Promega Corporation, Madison, WI, USA). Samples were analysed under a Bio-Rad Gel Doc 2000 (Bio-Rad Laboratories, Watford, Hertfordshire, UK). A Lambda DNA/HindIII (Promega Corporation, USA) DNA ladder was used to determine the size of the loaded PCR product.

#### 4.4.4. Quantification of mtDNA Damage

The range of the mtDNA damage after different types of stress was calculated as the relative amplification, compared to the undamaged control sample. Relative amplification of control samples was set to 1. It was assumed that the caused damage is Poisson distributed, which means that the likelihood for any damage in the 11 kb fragment is representative for the entire mtDNA genome. Accordingly, the relative amplification was converted into:

(*lesion* − *frequency*)/10 kbp = (0.908 × (−ln(*relative_amplification_of_treated_sample*)) (1)

### 4.5. Immunofluorescence Staining

Cells were seeded on cover slips (19 mm Ø, VWR, Lutterworth, Leicestershire, UK) in 12-well plates (IWAKI, Tokyo, Japan), treated as described above and fixed with 4% PFA. Cells were blocked and permeabilised with PBG-Triton (0.2% cold water fish gelatine, 0.5% BSA, 0.5% Triton X-100 in PBS), followed by 2 h of incubation with the primary antibody to telomerase reverse transcriptase, 1:2000, (Abcam/Epitomics UK, 32020). Afterwards cells were washed twice with PBG and incubated with the secondary antibody, IgG -goat-anti rabbit Alexa-Fluor 594, 1:2000 (Invitrogen, Inchinnan, UK) for 1 h, followed by PBS washes. Nuclear counterstain was performed with DAPI (CyStain UV Ploidy, Partec, Muenster, Germany) and the coverslips then mounted on Microscope Slides using Vectashield anti-fade mounting medium (Vector Laboratories, Peterborough, UK). Slides were examined with a Zeiss Axio Imager Z1 (Carl Zeiss Ltd., UK and AxioVision 4.8.2 (Carl Zeiss Ltd., Cambourne, UK) imaging software.

### 4.6. SDS PAGE and Western Blotting

Cell pellets were lysed using CHAPS buffer (Roche, Basel, Switzerland). Protein concentration was determined using a Bradford Assay (Bio Rad Laboratories, Hercules, CA, USA) and a Genova Fluorimeter (Jenway, Stone, Staffordshire, UK). 50 μg of protein was denatured with 2× Laemmli sample buffer at 95 °C for, then kept on ice. Samples and a PageRuler™ Prestained Protein Ladder (Fermentas, Elstree, Hertfordshire, UK) were loaded on a 12% polyacrylamide, then blotted on Amersham Hybond ECL nitrocellulose membranes (GE Healthcare, Amersham, UK). The membrane was blocked with 5% low fat milk in 1× TBST, following incubation with the primary antibody (see below) at 4 °C overnight. The next day, the membranes were washed 3× for 10 min with 1× TBST, then incubated with the secondary antibody (Goat polyclonal anti-rabbit HRP Abcam, UK or rabbit anti-mouse HRP, Abcam, UK) for 1 h at room temperature. The membranes were washed again with TBST. Chemiluminescence signals were detected using Amersham ECL™ Western blotting detection reagents (GE Healthcare, Amersham, UK) in a Fujifilm LAS-3000 imager (FUJIFILM Ltd., Broadstairs, Kent, UK). Densitometry analysis of band intensities was performed using AIDA Image Analyser V.4.13. To re-probe the membranes, bound antibodies were removed with Restore Western blot stripping buffer (Fisher Scientific Ltd., UK) washed with TBST buffer and blocked again with 5% low-fat milk. Primary antibodies used were: rabbit anti-beta-Tubulin (Abcam, Cambridge, UK), rabbit anti-catalase (Millipore, Burlington, MA, USA), rabbit anti-MnSOD (Abcam) and mouse FoxO3a (Abcam).

### 4.7. qRT-PCR

#### 4.7.1. RNA Isolation

Cell pellets were homogenised using QIAshredder columns (QIAGEN, Hilden, Germany) and total RNA was isolated using the RNeasy mini kit (QIAGEN, Hilden, Germany). RNA quality and amounts were determined with a NanoDrop^®^ ND-1000 spectrophotometer (Thermo Fisher Scientific, Waltham, MA, USA).

#### 4.7.2. cDNA Synthesis

One microgram (1 μg) of RNA and 1 μg random primers (Promega, Southampton, UK) were denatured at 75 °C for 7 min, then mixed on ice with 1× First Strand Buffer (Invitrogen, Inchinnan, UK), 0.2 μM DTT (Invitrogen, Inchinnan, UK), 10 μM dNTP (Roche Applied Science, Pennsberg, Germany), 40 U RNaseOUT™ RNAse Inhibitor (Invitrogen, Inchinnan, UK) and 200 U SuperScript^®^ III Reverse Transcrpitase (Invitrogen, Inchinnan, UK). cDNA synthesis was performed for 90 min at 42 °C followed by 95 °C for 5 min to stop the reaction.

#### 4.7.3. Real-Time PCR

Real time PCR was used to analyse gene-expression changes on RNA levels for SOD1 and SOD2 and Glutathione Peroxidase 3 (GPx3). Gene expression levels were normalised against the expression of GAPDH. 50 ng cDNA were used as template, together with 1× SYBR^®^ Green JumpStart™ Taq ReadyMix™ in 25 μL reactions with 0.2 μM primers (Table 1) and 1× ROX passive reference dye was added. PCR conditions were: an initial denaturation step of 94 °C for 6 min, followed by 30 cycles of 94 °C for 15 s, 60 °C for 45 s and 72 °C for 45 s. Melting curves were analysed between 60 °C and 95 °C with readings taken at every 1 °C on a StepOnePlus^TM^ real-time PCR system (Applied Biosystems, Warrington, UK).

### 4.8. Statistical Analysis

Graphs were generated and statistical analyses performed using Sigma Plot 13.0 (Systat Software Inc., San Jose, CA, USA) and GraphPad Prism 8.0. 1 (GraphPad Software Inc., La Jolla, CA, USA) and normality tested (Shapiro–Wilk test). Significance levels for multiple samples were calculated using a general linear test (two-way ANOVA) using the two different cell types and the different treatment conditions as factors without interactions between both factors. For the post-hoc test, Holm–Sidak for multiple pairwise comparisons was performed. Two samples (MRC-5 and hTERT overexpressing cells) at the same time point or the same concentration were compared using an unpaired Student’s *t*-test significance levels were determined as: * *p*-values ≤ 0.05, ** *p*-values ≤ 0.01, *** *p*-values ≤ 0.001.

## Figures and Tables

**Table 1:** Sequences of used primers for qRT-PCR.

Gene	5′ to 3′ Sequence
*SOD1* fw	agggcatcatcaatttcgag
*SOD1* rev	ccaaactcatgaacatggaatc
*MnSOD* fw	ctggacaaacctcagcccta
*MnSOD* rev	tgatggcttccagcaactc
*GPx3* fw	ggggacaagagaagtcgaaga
*GPx3* rev	gccagcatactgcttgaagg
*GAPDH* fw	tgcaccaccaactgcttagc
*GAPDH* rev	ggcatggactgtggtcatgag
